# Association of DNA methylation signatures with cognitive performance among smokers and ex-smokers

**DOI:** 10.18332/tid/168568

**Published:** 2023-08-21

**Authors:** Ping-Ching Hsu, Stacey B. Daughters, Michael A. Bauer, L. Joseph Su, Merideth A. Addicott

**Affiliations:** 1 Department of Environmental Health Sciences, Fay W. Boozman College of Public Health, University of Arkansas for Medical Sciences, Little Rock, United States; 2 Department of Psychology and Neuroscience, University of North Carolina at Chapel Hill, Chapel Hill, United States; 3 Department of Biomedical Informatics, University of Arkansas for Medical Sciences, Little Rock, United States; 4 Peter O'Donnell Jr. School of Public Health, UT Southwestern Medical Center, Dallas, United States; 5 Department of Physiology and Pharmacology, Wake Forest University School of Medicine, Winston-Salem, United States

**Keywords:** DNA methylation, cognitive performance, smoking, AHRR

## Abstract

**INTRODUCTION:**

Alterations in DNA methylation profiles have been associated with cancer, and can be influenced by environmental factors such as smoking. A small but growing literature indicates there are reproducible and robust differences in methylation levels among smokers, never smokers, and ex-smokers. Here, we compared differences in salivary DNA methylation levels among current and ex-smokers (at least 2 years abstinent).

**METHODS:**

Smokers (n=26) and ex-smokers (n=30) provided detailed smoking histories, completed the Paced Auditory Serial Addition Test (PASAT), and submitted a saliva sample. Whole-genome DNA methylation from saliva was performed, and ANCOVA models and a receiver operating characteristic (ROC) curve were used for the differences between groups and the performance of significant CpG sites.

**RESULTS:**

After controlling for race, age, and gender, smokers had significantly lower methylation levels than ex-smokers in two CpG sites: cg05575921 (AHRR) and cg21566642 (ALPPL2). Based on the ROC analyses, both CpGs had strong classification potentials (cg05575921 AUC=0.97 and cg21566642 AUC=0.93) in differentiating smoking status. Across all subjects, the percent methylation of cg05575921 (AHRR) and cg21566642 (ALPPL2) positively correlated with the length of the last quit attempt (r=0.65 and 0.64, respectively, p<0.001) and PASAT accuracy (r=0.29 and 0.30, respectively, p<0.05).

**CONCLUSIONS:**

In spite of the small sample size and preliminary research, our results replicate previously reported differences in AHRR hypomethylation among smokers. Furthermore, we show that the duration of smoking abstinence is associated with a recovery of methylation in ex-smokers, which may be linked to a reduced risk of smoking-associated diseases. The association with cognitive performance suggests that the hypomethylation of AHRR in saliva may reflect systemic exposure to cigarette-related toxicants that negatively affect cognitive performance, and should be validated in larger studies.

## INTRODUCTION

Epigenetics is the study of heritable changes in phenotype without actual changes in genotype, and DNA methylation is a common epigenetic signaling tool that cells use to regulate gene activation and expression. In mammals, the genome is composed of the DNA itself (the sequence of nucleotides) and a system of chemical modification (methylation) for instructing cells to express or not express certain genes. This is an important component in numerous cellular processes. DNA methylation occurs at the cytosine bases of eukaryotic DNA, which are converted to 5-methylcytosine by DNA methyltransferase enzymes (DNMTs). The altered cytosine residues are adjacent to guanine nucleotides, resulting in two methylated cytosine residues diagonal to each other on opposing DNA strands (CpG sites). Methylation of gene regulatory sequences, such as promotor or enhancers, represses expression. Demethylation can also occur. Understanding this methylation system is essential for deciphering how genes are expressed. Methylation plays a critical role in embryogenesis and tissue-specific differentiation, but methylation (and demethylation) continues to occur throughout the lifespan. Furthermore, an individual’s environment (e.g. nutrition, stress/trauma, toxicant exposure) has been theorized to cause epigenetic changes, such as DNA methylation. However, the details of this process and its physiological role are poorly understood.

Alterations in DNA methylation result in changes in gene expression and lead to the development of a spectrum of human diseases. Numerous environmental stressors have been shown to affect DNA methylation. Furthermore, interplay between DNA methylation and the environment is recognized as an important step in the response to environmental stimuli and the onset of disease. Importantly, DNA methylation signatures that result from an environmental exposure can be detected in saliva, buccal samples, and peripheral blood, making them great candidates for use as alternative exposure biomarkers. Chronic exposure resulting from cigarette smoking has been shown to be associated with extensive genome-wide differences in DNA methylation, in particular, the hypomethylation of the cg05575921 loci in the aryl hydrocarbon receptor repressor (AHRR) gene^1^. The AHRR gene regulates the aryl hydrocarbon receptor (AHR), which is the induction point for the xenobiotic pathway responsible for the degradation of environmental toxins commonly found in cigarettes. The hypomethylation of the AHRR gene shown in smokers likely represents increased AHR activation of this pathway from smoking exposure^2^ and smoking cessation is associated with more AHRR methylation^3^. Furthermore, the methylation status of cg05575921 CpG site from both whole blood and saliva have the same strong predicting power to predict smoking status and daily cigarette consumption^4^, making potential utility in ‘stress-free’ sampling in epidemiology studies to determine smoking exposure.

There is a growing literature on the association between DNA methylation profiles and changes in brain structure and function^5^. But to our knowledge, there has only been a single published study linking tobacco smoking-related differences in blood DNA methylation with cognitive function and other smoking-related health outcomes^6^. The study of Corley et al.^6^ referenced the smoking epigenetic scores (trained to predict pack-years of smoking) derived from the 230 CpGs in the Generation Scotland study, and further computed a DNA methylation score as a proxy for smoking exposure. They found that among older adults (aged 70 years), higher values of smokingrelated methylation scores were associated with the duration and intensity of smoking, lower cognitive function, and poorer structural brain integrity. Furthermore, the results indicated that methylation patterns accounted for more variance in smoking-related morbidities than phenotypic self-reports (e.g. smoking status and pack-years). Their results underscore the importance of DNA methylation as biomarkers of exposure^6^.

There are several possible mechanisms by which smoking cigarettes can negatively affect brain function and cognitive performance, in particular, toxicants in cigarette smoke produce oxidative stress and inflammation that can result in gray matter atrophy and reduced white matter integrity^7^. In particular, polycyclic aromatic hydrocarbons, a neurotoxic component of cigarette smoke that has been linked with cortical thinning^8^, induces the expression of the AHRR gene^9^. Smokers have been shown to have worse cognitive performance than non-smokers and ex-smokers^10^; however, to what extent cognitive performance can improve following smoking cessation is unknown. Studies indicate that ex-smokers with longer periods of cessation have DNA methylation patterns similar to never smokers^11^, suggesting that the methylation of smoking-affected CpG sites could serve as a biomarker for the duration and intensity of smoking among smokers, as well as the recovery from the harmful effects of smoking among ex-smokers. Thus, we hypothesized there would be an association between smoking-affected DNA methylation levels and cognitive performance. The aims of the current study are to test: 1) differences in the salivary DNA methylation levels among current and ex-smokers (at least 2 years abstinent) to validate previous findings from saliva samples; and 2) the association between salivary DNA methylation levels and cognitive performance.

## METHODS

### Participants

Current and former tobacco users were recruited from the Little Rock, AR community, as part of a larger neuroimaging study on distress tolerance. The sample included 26 current smokers, 30 ex-smokers (abstinent from all tobacco/nicotine for >2 years). Participants were aged 25–55 years; the lower age limit minimized potential age differences between groups (considering the ≥ 2-year abstinence criterion for ex-smokers), and the upper age limit reduced the likelihood of age-related changes in cognition. Smokers reported smoking ≥7 cigarettes/day for ≥2 years and had an expired breath carbon monoxide (CO) concentration of ≥5 ppm (Vitalograph Inc, Lenexa, KS). Smokers were excluded if they reported daily use of other tobacco products (e.g. little cigars or electronic cigarettes). Ex-smokers reported smoking ≥7 cigarettes/day for ≥2 years, but reported no use of tobacco or nicotine for ≥2 years, and had breath CO ≤5 ppm. The ex-smoker abstinence duration criterion minimized the likelihood of future relapse^12^. Participants were excluded if they met any of the following criteria: 1) reported serious health problems; 2) had a history of head trauma or neurological disorders; 3) currently met criteria for an Axis I psychiatric disorder (based on a MINI Neuropsychiatric Interview); 4) reported heavy drug use or problems with drugs or alcohol in the past 6 months (other than tobacco); 5) had a positive urine test for drugs (i.e. amphetamine, cocaine, methamphetamine, opioids, benzodiazepines, barbiturates) or breath test for alcohol; 6) reported using cannabis more than 4 days a week or more than 2 g per week; 7) were pregnant; 8) were using psychoactive medications other than first-line medications for depression (e.g. sertraline); 9) had less than a 9th grade education; 10) weighed more than 350 pounds (due to the weight limit of the MRI scanner); and 11) could not achieve 70% accuracy in the Paced Auditory Serial Addition Test (PASAT) easy practice task during eligibility screening. Participants were allowed to play the task up to 3 times to meet the criterion. Participants provided written informed consent and this study was approved by the University of Arkansas for Medical Sciences Institutional Review Board and conducted in accordance with the Declaration of Helsinki and relevant institutional guidelines and policies.

### Procedure

During eligibility screening, participants were administered a tobacco use history structured interview that assessed the current and lifetime use of cigarettes and other tobacco products, including the number and duration of cessation attempts, DSM-5 tobacco use disorder (TUD) severity scores, and the Fagerström test for nicotine dependence (FTND). Cigarettes per day, TUD, and FTND scores for ex-smokers were based on their past smoking behavior. If eligible, participants were scheduled for a 2-hour study session, including a 1-hour MRI scan and a 1-hour behavioral testing session. The results of the MRI and behavioral session will be reported elsewhere. Smokers were instructed to smoke immediately prior to the study visit and the time of their last cigarette was recorded. The PASAT was administered during the MRI scan. Following the MRI scan, participants submitted a saliva sample for DNA testing. Participants were allowed to opt-out of submitting a saliva sample and remain in the study. Participants were compensated by up to $178 for the completion of the study.

### Cognitive performance

Cognitive performance was assessed using the Paced Auditory Serial Addition Test (PASAT). The PASAT is a mental arithmetic task that requires participants to mentally sum numbers sequentially as they appear onscreen and select the correct sum from an array of options before the next number appears. The PASAT measures multiple functional domains, such as attention, working memory, information processing, and psychomotor performance. Participants had 1.5 sec to make a response. The percent of correct, on-time responses and the average reaction time for all on-time responses were the dependent variables.

### DNA extraction

Saliva samples provide a valid and convenient DNA source for exposure measurements. Saliva is a natural filtrate of blood that consist of leukocytes and endothelial cells, contains small molecules, metals, proteins, DNA, and reflects individual exposomes including diet, toxins, environmental chemicals, psychological health^13^, immune perturbation^14^, offering ‘stress-free’ sampling in epidemiology studies. Using the manufacturer’s protocol (PrepIT-L2P Oragene DNA Genotek Inc Kanata, Ontario, Canada) saliva sample tubes were inverted for gentle mixing and then incubated overnight in a C24 Incubator Shaker (New Brunswick Scientific, Edison, NJ) at 50°C and 85 rpm. A 500 μL aliquot of the saliva sample was transferred to 1.5 mL microcentrifuge tube (mct) for DNA extraction. A 20 μL aliquot of PrepIT-L2P was added to the sample which was mixed and then incubated on ice for 10 min. Samples were centrifuged to pellet impurities and the clear supernatant was transferred to a clean mct. To allow DNA precipitation 600 μL of 95–100% ethanol solution was added to each sample and allowed to incubate for 10 min at room temperature. The samples were centrifuged and the supernatant was discarded. The pellet was washed in a 250 μL aliquot of 70% ethanol solution. The DNA was eluted with 50 μL of 10 mM Tris-HCL, 1mM EDTA Buffer, pH 8.0 (Oragene Rockville, MD) and vortexed for 5 s. To ensure complete rehydration of the DNA pellet, the samples were allowed to incubate in a Thermomixer (Fisher Scientific, Hanover Park, IL) at 37°C and 300 rpm overnight. The concentration of DNA was quantified using Quant-iT PicoGreen ds DNA reagent kit (Invitrogen/Fisher Scientific, HANOVER PARK, IL) in duplicate using a SpectraMax M5 plate reader (Molecular Devices, SunnyVale, CA). Samples were diluted in TE to 40 ng/μL for a total of 800 ng and sent to the UAMS Genomics Core for methylation analysis.

### Infinium Methylation EPIC BeadChip analysis

Following bisulfite treatment of 1 μg genomic DNA using the EZ DNA Methylation kit (Zymo Research, Irvine, CA), the bisulfite-converted DNA was hybridized onto the Infinium Methylation EPIC BeadChip (Illumina, San Diego, CA), following the Illumina Infinium HD Methylation protocol in the Genomics Core Facility at UAMS. The Methylation EPIC BeadChip covers >850000 CpG sites, and has increased genome coverage of regulatory regions and higher reproducibility and reliability compared to previous versions^15^. Whole genome amplification, hybridization, staining and scanning steps for all samples were performed, the Illumina iScan SQ scanner was used to create images of the single arrays, and the intensities of the images were extracted using the Methylation module (v.1.9.0) of the GenomeStudio (v.2011.1) software (Illumina). Raw intensity data as IDAT files were imported into the ChAMP R package^11^ for the processing and analysis of the methylation arrays. The BMIQ algorithm was used in the normalization of the data. Probes on a blacklist of probes that are known to be cross-reactive were removed.

### Methylation data analysis

Differentially methylated probes were identified using the methylation pipeline in Partek Genomics Suite^TM^ 6.6 (Partek Inc., St. Louis, MO). Percent methylation values for each CpG site (β-values) and logit-transformed ratios of methylated to unmethylated probe intensities (M-values) were extracted for further analysis^16^. For pattern identification in DNA methylation, unsupervised analysis including unsupervised hierarchical clustering and Principal Component Analysis (PCA) were used. T-tests and chi-squared (χ^2^) tests were performed to evaluate differences between smokers and ex-smokers. Analysis of covariance (ANCOVA) adjusting for race, age, and gender with Fisher’s Least Significant Difference contrast method were used to assess the differentially methylated CpG sites univariately. The resulting p-values were adjusted for multiple testing with the false discovery rate (FDR) procedure of Benjamini and Hochberg, and significance was granted with FDR p<0.05 to identify significantly differential DNA methylation. Classical Receiver Operating Characteristic (ROC) curve analysis was used to evaluate the performance of a single CpG site as a biomarker.

### Pathway and gene ontology analysis

The genes corresponding to the significant loci were analyzed using Ingenuity Pathway Analysis^15^ (IPA) software (Ingenuity® Systems, www.ingenuity.com). IPA employs information obtained from the literature to assemble and extrapolate known interactions, signaling, as well as the relationships between the molecules. Biological functions and pathways were deemed statistically enriched when the FDR adjusted p-values <0.05 in Fisher’s exact test. Z-scores and p-values were used to predict potential upstream regulators. The p-value tests the probability of the genes in the gene list as being regulated by an upstream regulator by chance. Network analysis was generated *de novo* based on the mapped CpG sites to explore potential molecular events and mechanisms affected based on information obtained from the literature to assemble and extrapolate known interactions, signaling, as well as the relationships between these entities.

## RESULTS

### Demographic characteristics of the study participants

Table 1 shows the demographic characteristics and tobacco use histories for smokers and ex-smokers. Groups were similar in age, sex distribution, ethnicity, and years of education (all p>0.05). However, the smoker group had more Black participants (p=0.017). Smokers’ current FTND and DSM-5 TUD scores were similar to ex-smokers’ past scores (all p>0.05). Ex-smokers smoked more cigarettes per day (p=0.005), although the number of pack-years (i.e. smoking duration × number of packs per day) was similar between groups (p=0.19). As expected, ex-smokers had lower breath CO concentration (p<0.001) and the duration of their most recent quit attempt was longer than that of smokers’ (p<0.001). Ex-smokers quit smoking an average of 8.6 years ago (range: 2–21 years). PASAT accuracy (% correct) was lower among smokers compared to ex-smokers (p=0.023) and reaction time was longer among smokers compared to ex-smokers (p=0.011).

**Table 1:** Demographic characteristics and tobacco use histories of smokers and ex-smokers

*Characteristics*	*Smokers(N=26) mean ± SD*	*Ex-smokers(N=30) mean ± SD*	*Group differencep*
Age (years)	38.0 ± 9.6	40.3 ± 8.4	0.34
Sex (M/F)	15/11	12/18	0.19
Race (White/Black/Asian/Other)	17/6/1/2	29/0/0/1	χ^2^(3)=10.2 0.017
Ethnicity (non-Hispanic/Hispanic)	26/0	29/1	0.35
Years of education	13.9 ± 3.2	14.8 ± 2.4	0.24
FTND	5.0 ± 1.9	4.9 ± 2.2 (past)	0.96
DSM-5 TUD	5.9 ± 2.3	6.7 ± 2.2 (past)	0.23
Cigarettes per day	16.5 ± 5.9	22.6 ± 9.1 (past)	t(54)=2.9 0.005
Pack-years	21.5 ± 18.8	16.3 ± 10.1	0.19
Duration of most recent quit attempt (days)	96.0 ± 358.0	3144.2 ± 2006.3	t(54)=7.6 <0.001
Breath CO (ppm)	26.6 ± 15.5	2.2 ± 1.3	t(54)=8.6 <0.001
PASAT accuracy (% correct)	72.8 ± 11.9	80.0 ± 10.9	t(54)=2.3 0.023
PASAT reaction time (s)	0.935 ± 0.063	0.880 ± 0.090	t(54)=2.6 0.009

FTND: Fagerström test of nicotine dependence. DSM-5 TUD: DSM-5 tobacco use disorder symptom severity. PASAT: paced auditory serial addition test.

### Differential methylation analysis identified between smokers and ex-smokers

**Figure 1:**
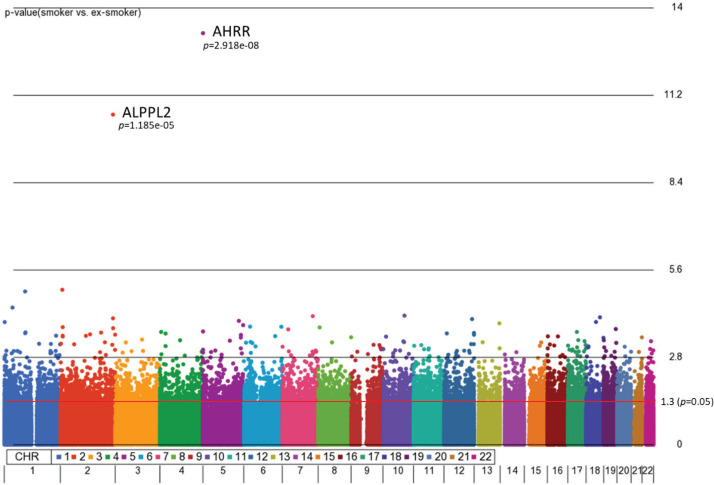
Manhattan plot of the chromosomal distribution of differentially methylated CpG sites between smokers and ex-smokers. The red horizontal line indicates between group difference (p<0.05)

First, we aimed to identify differentially methylated (DM) CpGs that can distinguish smoking status. From our 3-way analysis of covariance (ANCOVA) model controlling for race, age, and gender, 9808 CpG sites were significantly different between smokers and ex-smokers with unadjusted p<0.05 and 122 CpGs (p<0.001), but only two CpGs passed multiple-testing adjustments (Bonferroni p<0.05, Figure 1). The mean F-ratio for each covariate was computed to show the significance of different sources of variation in the entire data in the ANCOVA model (Supplementary file Figure 1). Based on the ANCOVA model, gender and race contributed little variation to the model compared to random error, while age provided slightly higher F-ratio in the model which was adjusted in the ANCOVA model.

**Figure 2:**
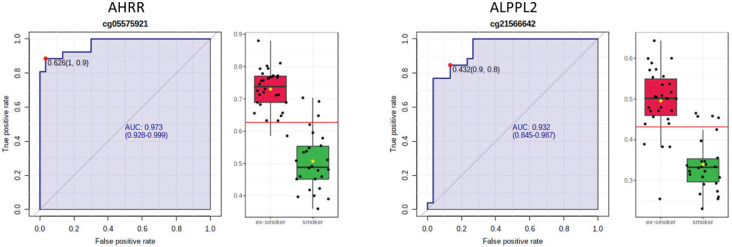
ROC curve analysis of CpG sites on (a) AHRR (cg05575921) and (b) ALPPL2 (cg21566642). The sensitivity (true positive rate) is on the y axis, and the specificity (one minus the false positive rate) is on the x axis with the area under the curve (AUC, in blue) of 0.973 (95% CI: 0.928–0.999) for AHRR and 0.932 for ALPPL2 (95% CI: 0.845–0.987). Box plots represent the percent methylation in β values (y axis) for (a) AHRR and (b) ALPPL2 between ex-smokers and smokers. The red horizontal line indicates the optimal cutoff

AHRR, 6p21.33, and F2RL3 have been found to be associated with smoking status in several studies^17^. Among the 9808 significant CpGs (p<0.05), AHRR (9 CpGs), 6p21.33 (1 CpG), and F2RL3 (1 CpG) were significantly lower in the methylation levels among smokers compared to ex-smokers (Supplementary file Table 1). The two CpG sites were cg05575921, which binds to the North Shore of AHRR gene on chromosome 5, and cg21566642, which binds to the CpG island of ALPPL2 gene on chromosome 2. Both CpG sites were hypermethylated among ex-smokers and hypomethylated among smokers (cg05575921: fold change=1.4; unadjusted p=6.67×10^-15^, Bonferroni p=3.02×10^-9^; cg21566642: fold change=1.5; unadjusted p=1.4×10^-10^, Bonferroni p=6.4×10^-5^). The area under the curve (AUC, Figure 2) for cg05575921 is 0.97 and for cg21566642 it is 0.93 in ROC analysis (Figure 2), both represent high sensitivity and specificity to accurately classify the two groups.

We further computed Pearson’s correlations to examine the correlation of the two CpG sites with cognitive performance variables. The results in Table 2 show the correlations and partial correlations between percent methylation of cg05575921 (AHRR) and cg21566642 (ALPPL2) with smoking history and cognitive performance. Percent methylation of cg05575921 (AHRR) was positively correlated with abstinence duration (r=0.65; p=4.0×10^-7^, Supplementary file Figure 2) and PASAT % accuracy (r=0.29; p=0.03), and negatively correlated with pack-years in the raw model (r= -0.29; p=0.03); the correlation of percent methylation of cg05575921 (AHRR) and PASAT % accuracy decreased slightly when controlling for age, race, and gender (r=0.26; p=0.06). The cg21566642 (ALPPL2) was also positively correlated with abstinence duration (r=0.64; p= 4.4×10^-7^, Supplementary file Figure 2) and PASAT % accuracy (r=0.30; p=0.02), and negatively correlated with pack-years (r= -0.36; p=0.006) in the raw model; the correlation of percent methylation of cg21566642 (ALPPL2) and with abstinence duration increased slightly when controlling for age, race, and gender (r=0.0.74; p=4.9×10^-9^).

**Table 2:** Correlations and partial correlations among all subjects between percent methylation of cg05575921 and cg21566642, smoking history, and cognitive performance

	*AHRR cg05575921*		*ALPPL2 cg21566642*	
	Model 1	Model 2	Model 1	Model 2
Abstinence duration (days)	0.65**	0.72**	0.64**	0.74**
Pack-years	-0.29*	-0.33*	-0.36**	-0.36**
PASAT % accuracy	0.29*	0.26	0.30*	0.29*
PASAT reaction time (s)	-0.22	-0.23	-0.22	-0.21
FTND	-0.11	-0.12	-0.16	-0.17
DSM TUD	0.06	0.08	0.07	0.07

Model 1: raw model. Model 2: controlling for age, race, and gender.

* p<0.05.

** p<0.001.

**Figure 3:**
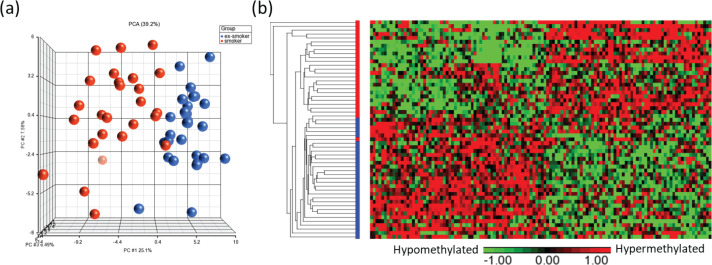
Visualization of the top CpG sites (p<0.001) in (a) principal component analysis and (b) hierarchical clustering distinguishing methylation levels between smokers and ex-smokers. To determine the differences in the global methylation profiles between smokers and ex-smokers, a 3-way ANCOVA model controlling for race, age, and gender was used. There were 9808 CpGs (p<0.05) and 122 CpGs (p<0.001) differentially methylated CpGs between smokers and ex-smokers

Principal component analysis (PCA) modeling of the top CpGs (p<0.001) demonstrated separation of methylation profiles between smokers and ex-smokers (Figure 3a), and hierarchical clustering were performed based on the top 122 significant CpG sites which shows clustering of the samples into two groups based on the smoking status (Figures 3a and 3b).

### IPA pathway analysis

**Figure 4:**
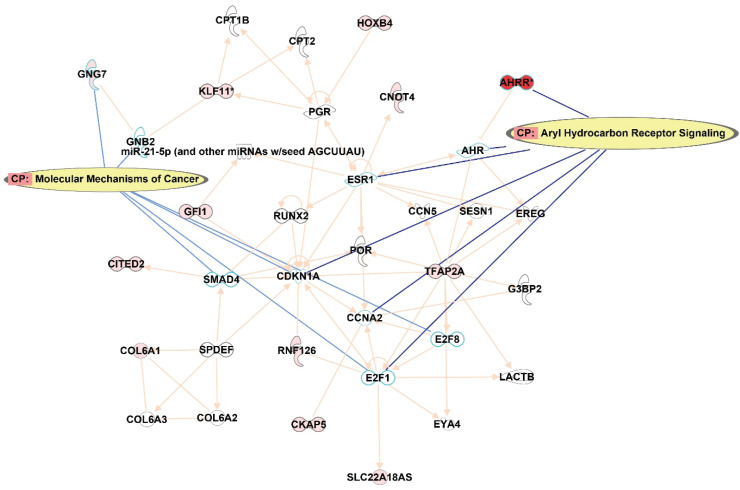
Network analysis generated based on the top 122 CpGs sites that differentiated smokers and ex-smokers from the Ingenuity pathway analysis. Red nodes represented CpG sites mapped with increased methylation among ex-smokers compared to smokers in this study. Aryl hydrocarbon receptor signaling and molecular mechanisms of cancer were the top canonical pathways involved in the network

In order to understand the potential impact on the pathways, IPA was queried from the top 122 CpG sites that were differentially methylated (p<0.001) between smokers and ex-smokers. This analysis considered direct relationships, focusing on interaction networks based on experimentally observed human tissues or cell lines. Network analysis was generated *de novo* based on the mapped CpG sites to explore potential molecular events and mechanisms affected. The network affected by the DM CpGs (Figure 4) was associated with cancer, organismal injury and abnormalities, cardiovascular disease, with the top canonical pathways involved including aryl hydrocarbon receptor signaling and molecular mechanisms of cancer.

## DISCUSSION

The smokers and ex-smokers in this sample population had different patterns of DNA methylation, especially on the cg05575921 (AHRR), cg21566642 (ALPPL2) CpG sites. The percent methylation of both CpG sites negatively correlated with pack-years, and positively correlated with cognitive performance accuracy. The duration of smoking abstinence is associated with a recovery of methylation in ex-smokers, which may be linked to a reduced risk of smoking-associated diseases. Of the top 122 significantly different CpG sites, a pathway analysis indicated an association with cancer and cardiovascular disease.

Tobacco smoking, which is a leading cause of disease and death worldwide, is associated with extensive genome-wide changes in DNA methylation. A small but growing literature indicates that there are reproducible and robust differences in methylation among smokers, never smokers, and ex-smokers. One large-scale study examined DNA methylation among smokers and never smokers and identified 187 replicable CpG sites with differential methylation levels^18^. Among ex-smokers, most of the CpG sites with smoker/never-smoker differences had DNA methylation comparable to never smokers, and ex-smokers’ methylation varied according to time since quitting. However, 13 of the 187 replicable sites remained hypomethylated in ex-smokers, albeit to a lesser extent than smokers^18^.

Zeilinger et al.^18^ evaluated whole blood DNA of current, former and never smokers from 1793 KORA participants using the Illumina 450K BeadChip, and identified cg05575921 (AHRR) with having the highest level of changes in DNA methylation associated with tobacco smoking, together with two ALPPL2 CpG sites (cg21566642 and cg01940273). McCartney et al.^19^ reported 234 CpG sites that were differentially methylated between 102 smokers and 418 non-smokers, including four CpGs: cg23079012 at chromosome 2p25.1, cg05575921(AHRR), cg06644428 and cg21566642 (both mapped to ALPPL2 at chromosome 2) that were also among the top 122 CpGs identified in our study. Furthermore, 6p21.33 and F2RL3 have been found to be associated with smoking status in several studies^17^, and were significantly lower (p<0.05) among smokers compared to ex-smokers in our study.

Lastly, a meta-analysis study of DNA methylation across 16 cohorts described 2623 CpG sites with differential methylation between smokers, never smokers, and ex-smokers, suggesting a potentially stable biomarker of lifetime exposure to tobacco smoke^20^. This idea was tested in a subsequent large-scale study using cross-sectional and longitudinal data^21^. A smoking methylation polyepigenetic score (a composite measure of 2623 smoking-related DNA methylation sites) was applied to participants’ DNA methylation profiles and revealed a step-wise increase in scores from never smokers, to ex-smokers, and current smokers. Furthermore, scores increased longitudinally according to an increasing number of pack-years and scores declined longitudinally among individuals who quit smoking^21^. Unfortunately due to small sample size, only two CpGs identified in the present study passed multiple-testing adjustments (Bonferroni p<0.05, Figure 1). Therefore, we did not proceed with the development of polygenic scores in this study. Altogether, these studies and others indicate there are reliable methylation changes related to tobacco smoking and smoking cessation.

In particular, smoking reliably results in the hypomethylation of the cg05575921 loci in the AHRR gene in both blood and saliva,^4^ and it takes between 2 and 14 years for ex-smokers’ DNA methylation levels to return to non-smoker levels^11^. This methylation pattern in blood and saliva/buccal cells is a reproducible and highly robust biomarker of exposure to accurately predict smoking status and smoking intensity^4^ across large-scale studies^22^. Another large-scale study examined DNA methylation among female smokers, never smokers, and ex-smokers and replicated previous work, including hypomethylation of AHRR among smokers and ex-smoker methylation returning to never-smoker baselines in relation to time since quitting. In addition, these results indicated that hypo/hypermethylation patterns in smokers were correlated with the heaviness of smoking^1^. Smoking cessation has been associated with increased methylation of AHRR^3^. Methylation of AHRR also predicts exposures to polycyclic aromatic hydrocarbons (PAHs)^23^, airborne particulate matter^24^, post-traumatic stress disorder in non-smokers^25^, and residence in a disadvantaged neighborhood^26^. While we do not suggest that the methylation of the AHRR gene has any direct or causal effects specifically on brain function or cognitive performance, the associations between PASAT performance and the reduction in methylation levels among ex-smokers may be indicative of a broad, physiological recovery from the detrimental effects of smoking.

Hypomethylation of the aromatic hydrocarbon receptor repressor (AHRR) CpG site (cg05575921) has been increasingly used as a robust biomarker of smoking history^3^. In this study, AHRR CpG site cg05575921 is correlated with abstinence duration and PASAT % accuracy, with the potential to capture lifetime smoking history that the traditional biomarker of exposure (cotinine, 3-hydroxycotinine) cannot achieve for ex-smokers (Supplementary file Figure 2). The PASAT is a widely used measure of cognitive function, and engages a variety of processes such as working memory, sustained attention, psychomotor reaction time, and mental arithmetic. The value of comparing smokers to ex-smokers is to inform the extent to which smoking abstinence can be linked to physiological benefits. While there may have been pre-existing differences between groups in cognitive performance, the correlation with DNA methylation biomarkers suggests that better performance is linked to the duration of smoking abstinence. The group differences in cognitive accuracy reported here align with both cross-sectional and longitudinal cohort studies that have indicated slower or poorer cognitive performance among smokers compared to ex-smokers and non-smokers^27^. Chronic cigarette smokers typically perform worse on measures of attentional control, inhibition, memory, and information processing speed compared to age-, sex-, and education level-matched non-smokers^10^. However, the extent to which smoking cessation can potentially reverse or prevent cognitive-associated damage from smoking is unclear, as there are few published studies comparing smokers to ex-smokers. One such study indicated that, among older adults (aged >67 years) enrolled in a smoking cessation trial, smokers who were able to maintain abstinence for 2 years had better cognitive scores (adjusted for baseline) at follow-up than smokers who did not quit^28^. The cognitive scores of smokers who did not quit worsened over the 2-year follow-up period. Cigarette smoke consists of numerous compounds associated with brain toxicity that can result in inflammation, atherosclerosis, white matter hyperintensities, and brain atrophy, all of which can negatively impact cognitive function over time^7^. While some evidence suggests that nicotinic receptor systems return to non-smoker levels following smoking cessation and ex-smokers demonstrate better cognitive performance compared to smokers, the extent to which tobacco cessation mitigates the structural and functional effects of tobacco on the brain is largely unknown. Recent review on the impact of tobacco on cognition suggested chronic smoking reduces hippocampus-dependent learning, bioelectric dysfunction at the cortical and subcortical levels, lower motor cortex activation, and that any level of prenatal tobacco exposure will affect children’s development^29^. In this pilot study, the PASAT accuracy was lower among smokers compared to ex-smokers, and reaction time was longer among smokers compared to ex-smokers. Nonetheless, it should be also mentioned that the main limitation in our study was small sample size for each group, which limits statistical precision in the analysis models.

Saliva is a natural filtrate of blood that consist of leukocytes and endothelial cells, contains small molecules, metals, proteins, DNA, and reflects individual exposomes including diet, toxins, environmental chemicals, psychological health, immune perturbation, offering stress-free’ sampling in epidemiology studies. Several validation studies have demonstrated up to 88.5% concordance between DNA methylation profiles from matched whole blood and saliva-derived DNA^30^. Although it has limited capability to study cell-type specific epigenetic mechanisms linked to disease etiology, saliva samples provide a valid and convenient DNA source for exposure measurements especially for large-scale epidemiology studies and underserved minority populations. Philibert et al.^6^ compared the prediction power of AHRR methylation between DNA from saliva and blood with convincing results of AUC with 0.971 from saliva and 0.995 from blood in predicting smoking status using ROC. DNA methylation markers account for more variance in smoking-related morbidities than phenotypic self-reports. In this study, we intend to highlight DNA methylation signatures from saliva as smoking exposure markers with the potential to capture lifetime smoking history that the traditional biomarker of exposure (cotinine, 3-hydroxycotinine) cannot achieve for ex-smokers. The correlations of those smoking exposure markers with the change of cognitive performance between smokers and ex-smokers can serve as a valuable complement to self-reported smoking history because such biomarkers are not affected by recall bias and may be more sensitive measures of smoking-related disease risk.

## CONCLUSIONS

Altogether, the literature has shown reproducible epigenetic biomarkers of smoking exposure. Furthermore, it suggests there is a potential for DNA methylation to become a powerful new tool for investigating the molecular mechanisms by which smoking affects cognitive performance and physical health in spite of the small sample size and preliminary research. Future research with larger sample sizes is needed in order to validate the DNA methylation CpG sites to serve as biomarkers of long-term smoking exposure and smoking cessation, with the potential to complement existing methods such as self-reported smoking histories and nicotine metabolite levels.

## Data Availability

The data supporting this research are available from the authors on reasonable request.
