# Supplementary material for: Association of DNA methylation signatures with cognitive performance among smokers and ex-smokers
Source: Tob Induc Dis. 2023 Aug 21;21:106. doi: 10.18332/tid/168568 (PMC10405227; doi:10.18332/tid/168568)
Supplement: Supplementary file 1 [file TID-21-106-s1.pdf]

Supplementary file Figure 1. Source of variation in the ANCOVA model.

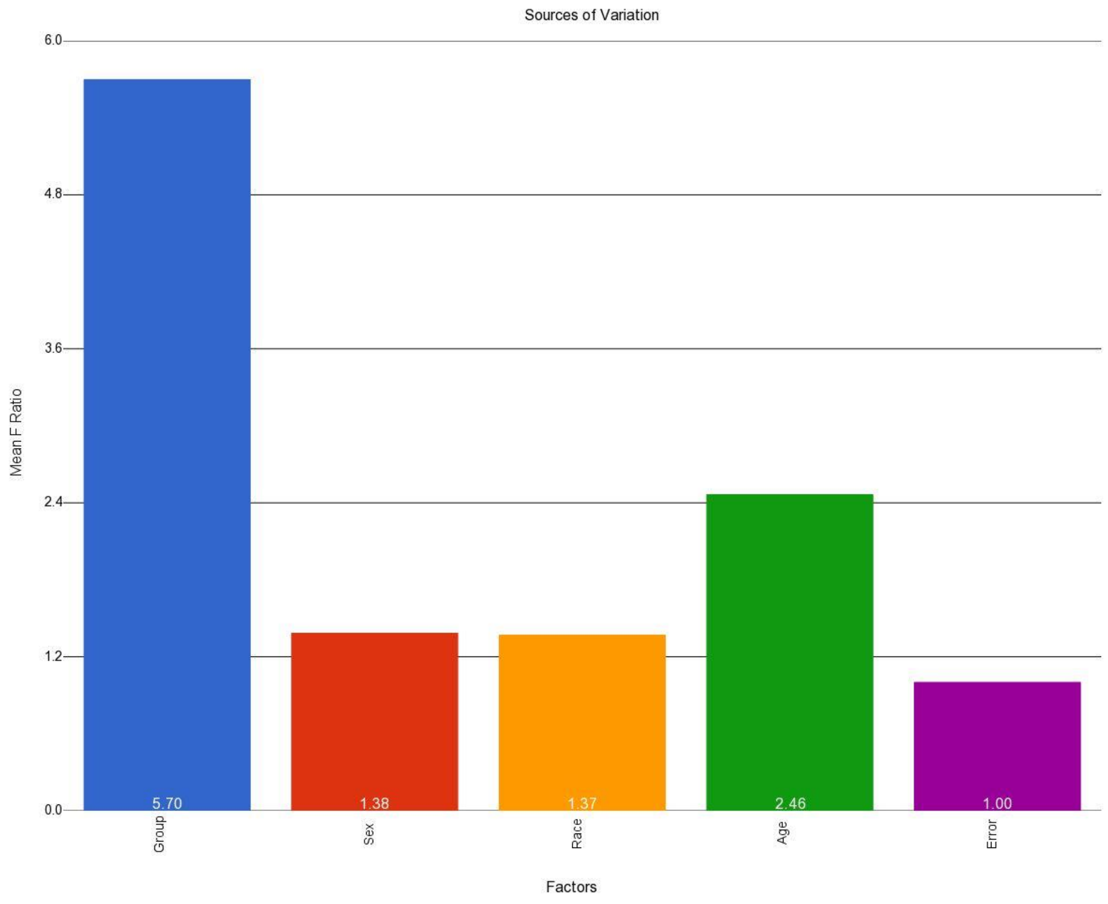

Supplementary file Figure 2. Correlation of (a) cg05575921 and (b) cg21566642 with abstinence duration in days. Red dots represent smokers while blue dots represent ex-smokers.

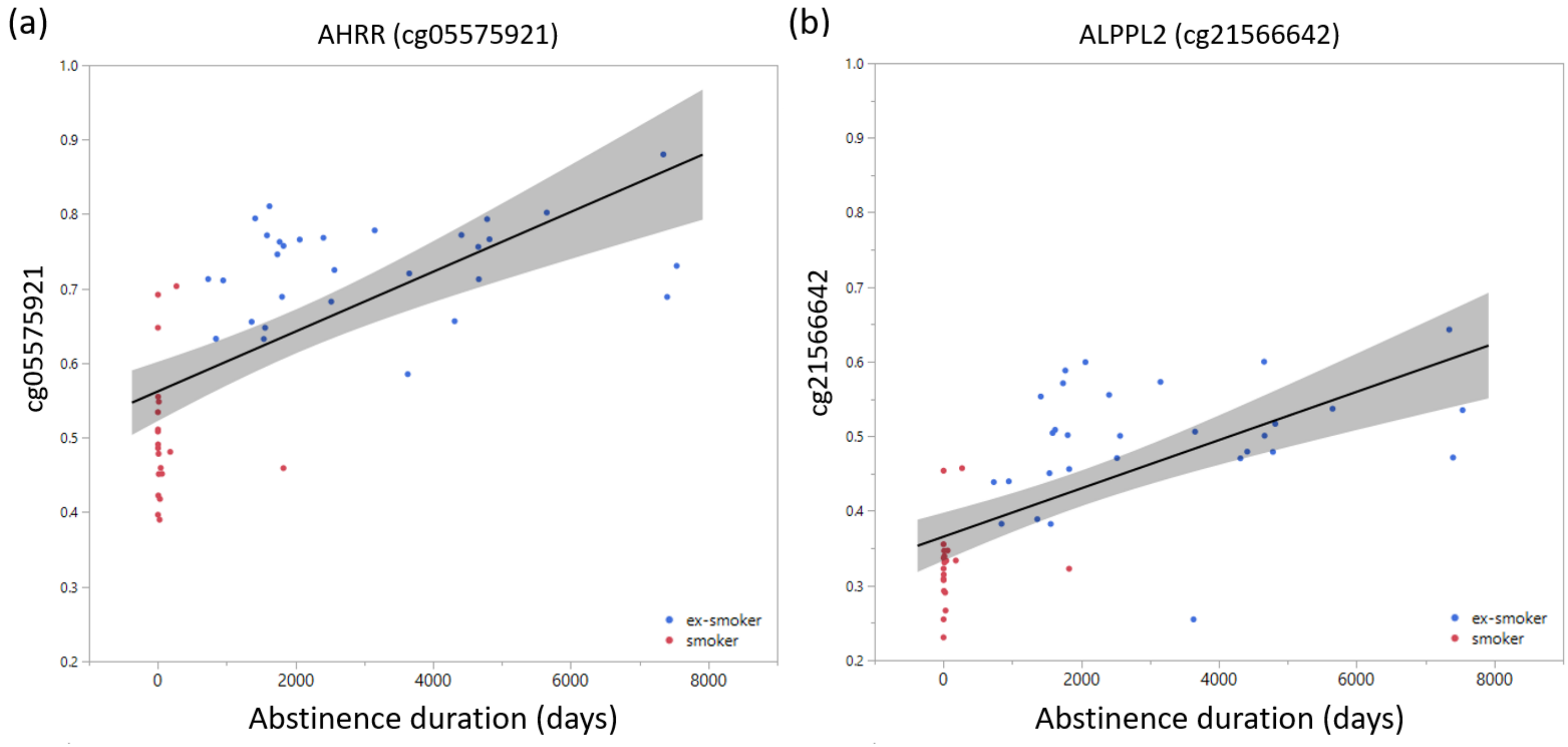

Supplementary file Table 1. Other widely validated smoking related methylation loci identified in this study.

Probeset ID	Gene Symbol	CHR	Relation_to_UCSC_CpG_Island	UCSC_RefGene_Group	*p* (smoker vs. ex-smoker)
cg05575921	AHRR	5	N_Shore	Body	6.43E-14
cg25648203	AHRR	5	Open sea	Body	2.34E-04
cg14753356	6p21.33	6	Open sea		0.001
cg24090911	AHRR	5	Open sea	Body	0.001
cg11554391	AHRR	5	Island	Body	0.001
cg01970407	AHRR	5	S_Shore	Body	0.002
cg06036945	AHRR	5	Open sea	Body;Body	0.004
cg09584122	AHRR	5	Open sea	Body	0.013
cg14982043	AHRR	5	Open sea	Body;3'UTR	0.029
cg07137034	AHRR	5	Island	Body	0.042
cg21911711	F2RL3	19	N_Shore	TSS1500	0.047

© 2023 Hsu P.C. et al.
